# Analysis of parasite communities and potentially pathogenic parasites in wild takin (*Budorcas taxicolor*)

**DOI:** 10.3389/fvets.2025.1555400

**Published:** 2025-03-24

**Authors:** Xiangwen Zeng, Ruiguo Liu, Rongyan Luo, Bingying Li, Jianing Liu, Zhiguo Li, Weichen Wang, Lijun Cai, Mingfu Li, Mei Xiao, Xiaoping Ma

**Affiliations:** ^1^Key Laboratory of Animal Disease and Human Health of Sichuan Province, College of Veterinary Medicine, Sichuan Agricultural University, Chengdu, China; ^2^Management Office of Tangjiahe National Nature Reserve, Qingchuan, Sichuan, China

**Keywords:** *Budorcas taxicolor*, wild animal, potentially pathogenic parasites, plant-parasite correlations, 18S rRNA amplicon sequencing

## Abstract

**Background:**

The endangered takin (*Budorcas taxicolor*) faces health risks from parasitic infections, including gastrointestinal helminths and protozoa. While previous studies have explored its gut microbiome, research on parasites remains limited. Investigating parasite diversity and its effects on takin health is crucial for effective conservation.

**Methods:**

59 fecal samples were collected from the Sichuan Tangjiahe National Nature Reserve, China, across elevations of 1,100–2,500 meters. The samples were categorized into four groups based on location. DNA was extracted using the CTAB method, amplified for 18S rRNA, and sent for sequencing.

**Results:**

Analysis of takin fecal samples from Tangjiahe revealed significant differences in eukaryotic communities among the RA, RB, RC, and RD groups. Several potentially pathogenic helminths and protozoa were identified, including *Oesophagostomum*, *Dictyocaulus*, *Entamoeba*, and *Eimeria*. Some parasites, such as *Aelurostrongylus*, exhibited high abundance and widespread distribution. While they are harmless to takins, they are capable of infecting other animals. The correlation between parasite abundance and plant community composition suggests that certain plants may act as vectors facilitating parasite transmission.

**Conclusion:**

This study highlights the potential impact of nematodes and protozoa on the health of the Sichuan takin population in Tangjiahe, while also examines the relationship between the dietary composition of takins and parasitic infections. This has important ecological and practical implications for wildlife conservation and health management.

## 1 Introduction

The takin (*Budorcas taxicolor*), a bovid species within the order Artiodactyla, is listed as endangered by the IUCN. It inhabits dense forests across the eastern Himalayas and parts of southwestern and northwestern China, with a geographical range extending to Bhutan, northeastern India, and northern Myanmar. The species is typically found at elevations ranging from 1,500 to 4,500 meters (1, 2). Its distinctive morphology and habitat have made it a focal species in ecological and evolutionary studies.

The Sichuan takin (*Budorcas taxicolor tibetana*) distributed across the entire Tangjiahe region, exhibit distinct seasonal vertical migration patterns, making two migrations annually. This seasonal variation in their activity range not only reflects the Sichuan takin’s habitat adaptation but is also closely linked to factors such as food resource distribution and climate change (3–5). As an omnivorous herbivore, the takin feeds on over 160 plant species, primarily consuming understory shrubs and herbaceous plants (6). Long-term field monitoring in the Tangjiahe Nature Reserve shows that while the takin population remains generally healthy, a few individuals still die each year, with most exhibiting lung lesions. This phenomenon has raised further concerns regarding the health threats to takins, particularly the potential role of parasites in these health issues. Therefore, investigating the parasitic threats faced by takins is a crucial part of conservation efforts.

Parasitic diseases are among the most prevalent health threats to wildlife, significantly impacting their survival and quality of life (7). In wild herbivorous ruminants, gastrointestinal helminths—such as tapeworms, roundworms, and flukes—constitute the most common parasitic taxa (8). Protozoa also play critical roles in ruminant health, with commensal species like rumen ciliates and flagellates contributing to fiber digestion and nutrient absorption (9). However, pathogenic protozoa, including *Eimeria* and *Theileria*, pose significant health risks. Our previous studies have primarily examined the composition and functional roles of the gut microbiome in takins, emphasizing the diversity of gut-associated fungi and bacteria and their potential implications for host health (10, 11). However, compared to microbiome studies, research on parasites remains relatively underexplored. Given the potential significant role that parasites may play in host health issues, it is particularly important to investigate their community composition and the relationship between parasites and host health.

Fecal samples provide invaluable insights into wildlife health, offering a non-invasive means to study diet, gut microbiota, and potential parasitic infections. Recent advancements in high-throughput sequencing (HTS) technologies have enabled the efficient detection of parasites in environmental samples, facilitating the assessment of parasite diversity and epidemiological patterns (12). Leveraging these tools, we conducted 18S rRNA amplicon sequencing on 59 fecal samples collected from wild takins in Tangjiahe. This molecular approach allowed a comprehensive analysis of parasite composition and abundance, providing critical data for health assessments and informing conservation strategies for this endangered species.

## 2 Materials and methods

### 2.1 Sample collection

Samples were collected from the Sichuan Tangjiahe National Nature Reserve, China (32°59′N, 104°77′E). In November 2022, we collected a total of 59 takin fecal samples from the Tangjiahe region, spanning an elevation range of 1,100–2,500 m. Based on the sampling locations, the samples were categorized into four groups: RA (Motianling), RB (Baiguoping), RC (Baixiongping), and RD (Caijiaba). To ensure sample integrity during collection, fecal samples were handled with sterile tubes to avoid surface contact, and separate tools were used for each sample to prevent cross-contamination. After collection, the samples were immediately stored on dry ice or in a − 20°C freezer. Within two hours, they were transported to the laboratory and transferred to liquid nitrogen for long-term preservation and subsequent analysis (10, 11).

### 2.2 DNA extraction

Following the manufacturer’s protocol (Solarbio, Beijing, China), DNA was extracted from the fecal samples using the cetyltrimethylammonium bromide (CTAB) method, with nuclease-free water serving as the negative control. The extracted DNA was eluted in 50 μL of elution buffer, stored at −80°C, and subsequently sent to LC-Bio Technology Ltd. (Hangzhou, Zhejiang, China) for PCR amplification.

### 2.3 PCR and NGS sequencing

We amplified the V9 region of the 18S rRNA gene, which is suitable for biodiversity assessments (13), using the specific primers 1391f (5’-GTACACACCGCCCGTC-3′) and EukBr (5’-CTTCTGCAGGTTCACCTAC-3′) (14). The PCR reactions were carried out in a total volume of 25 μL, consisting of 25 ng template DNA, 12.5 μL PCR premix, 3 μM of each primer, and ddH₂O to make up the final volume. The PCR cycling conditions included an initial denaturation at 98°C for 30 s, followed by 32 cycles of denaturation at 98°C for 10 s, annealing at 54°C for 30 s, and extension at 72°C for 45 s, with a final extension at 72°C for 10 min.

The PCR products were visualized using 2% agarose gel electrophoresis, then purified and quantified using AMPure XT beads (Beckman Coulter Genomics, Danvers, MA, USA) and a Qubit fluorometer (Invitrogen, Waltham, MA, USA). The size and concentration of the amplicon libraries were assessed with an Agilent 2,100 Bioanalyzer (Santa Clara, CA, USA) and the Illumina Library Quantification Kit (Waltham, MA, USA). High-throughput sequencing was performed on the NovaSeq PE250 platform (Illumina, San Diego, CA, USA).

### 2.4 Data analysis

Following sequencing, raw data (RawData) were obtained and processed to generate high-quality clean data (CleanData). Paired-end reads were merged using overlap, followed by quality control and chimera filtering. High-resolution amplicon sequence variant (ASV) tables and representative sequences were generated using the DADA2 algorithm, with random normalization applied to ensure data comparability across samples.

Taxonomic annotation was conducted by aligning ASV sequences to the NCBI database using the BLAST algorithm. Alpha diversity was assessed using the Chao1 and Shannon indices, while beta diversity was analyzed through principal coordinate analysis (PCoA) to evaluate community structure differences. All statistical analyses and visualizations were performed in R (v3.5.2). All raw sequence data were uploaded to the National Center for Information Biotechnology Information Search^1^ under the registration number PRJNA1201350.

## 3 Results

### 3.1 Sequencing date

From 59 samples collected in the Tangjiahe region, we obtained 5,129,427 raw 18S rRNA sequences. Following sequence assembly, quality control, and filtration, a total of 4,978,387 high-quality reads were retained, with an average sequencing depth of 84,379.4 reads per sample. After removing non-target sequences (e.g., bacterial reads), we identified 2,681 unique amplicon sequence variants (ASVs). Based on sampling locations, the samples were grouped into four regions: RA, RB, RC, and RD. The analysis revealed 540, 583, 161, and 556 ASVs unique to each group, respectively, with 224 ASVs shared across all four regions (Supplementary Figure S1).

### 3.2 Alpha diversity analysis

**Figure 1:**
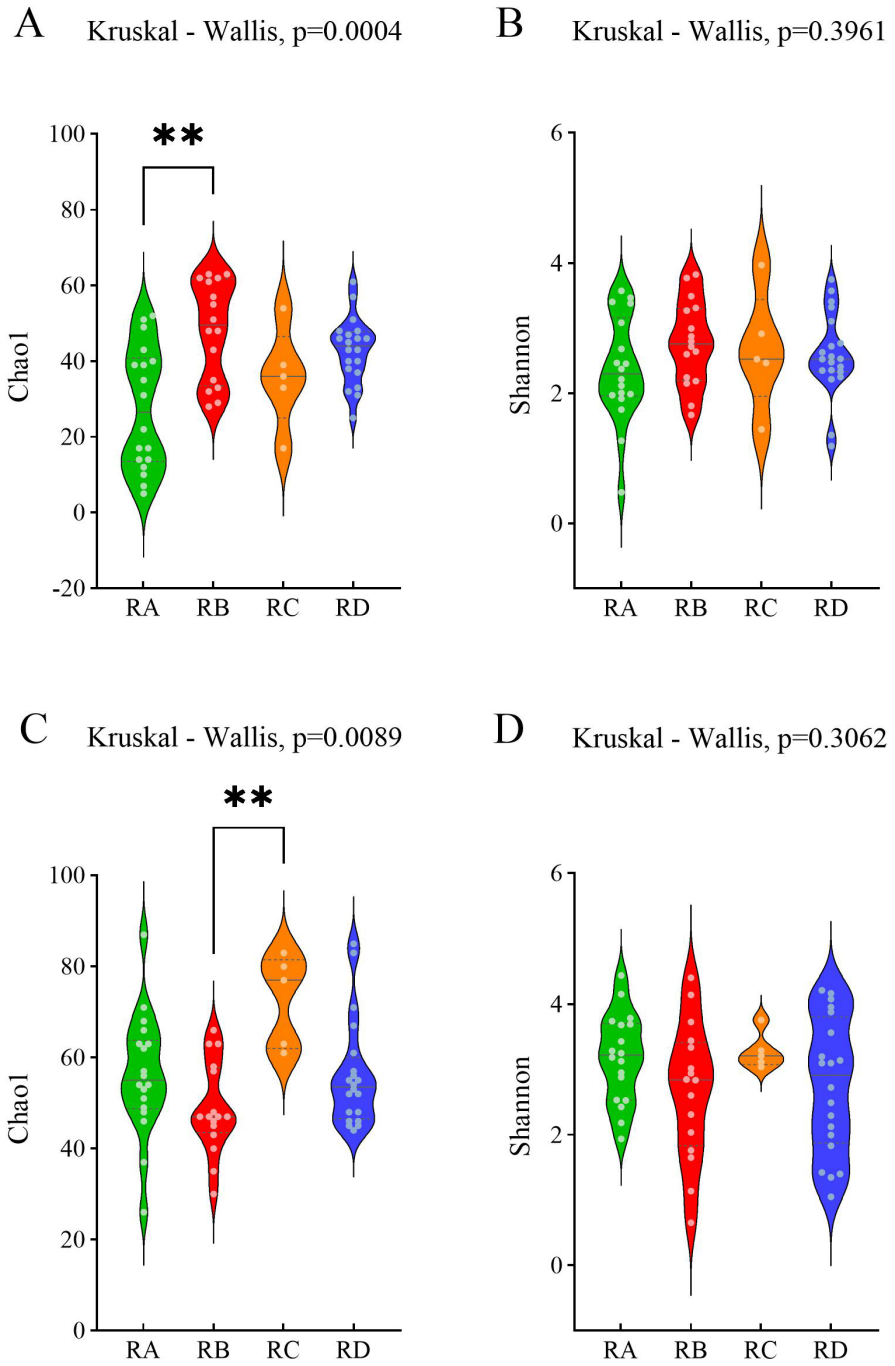
Violin plots illustrating the distribution of Chao1 and Shannon indices across different groups. **(A)** Chao1 index of plants. **(B)** Shannon index of plants. **(C)** Chao1 index of parasites. **(D)** Shannon index of parasites.

We assessed the abundance and diversity of eukaryotes in the fecal samples of Sichuan takin across four regions using the Chao1 and Shannon indices. The results indicated no significant differences in the abundance or diversity of eukaryotes among the regions (Supplementary Figure S2). Additionally, we analyzed the abundance and diversity of plants in the fecal samples to infer dietary patterns of *Budorcas taxicolor* in different regions. Alpha diversity analysis revealed that plant abundance in region RA was significantly lower than in the other three regions (Figure 1A), while plant diversity showed no significant variation across regions (Figure 1B). Similarly, we compared the Chao1 and Shannon indices of parasites (including helminths and protozoa) across the regions. Parasite abundance and diversity did not differ significantly among the groups, except for the abundance in group RB, which was significantly lower than that in group RC (Figures 1C,D).

### 3.3 Beta diversity analysis

**Figure 2:**
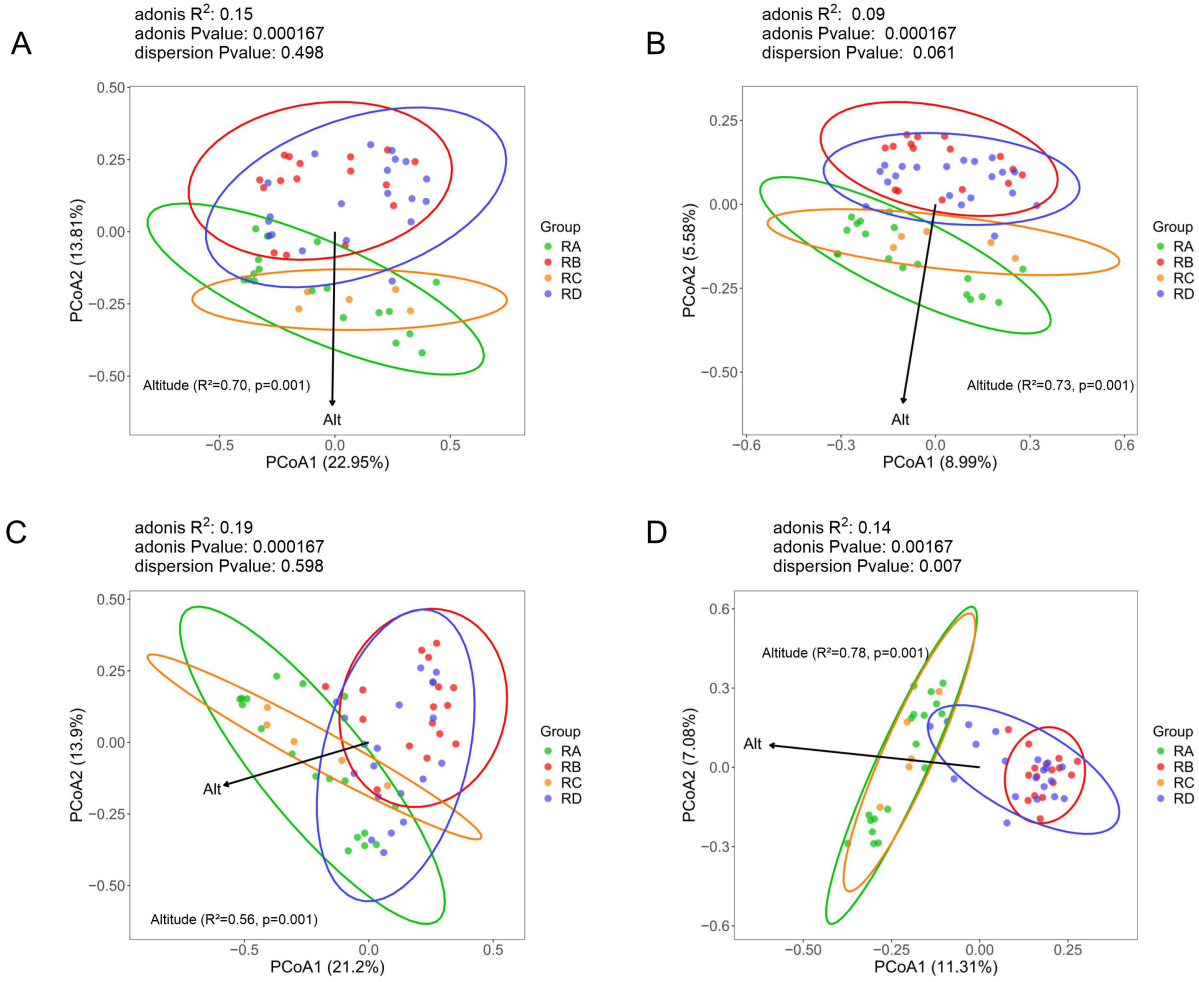
PCoA analysis based on Bray-Curtis and Binary Jaccard dissimilarities. **(A)** All eukaryotes (Bray-Curtis). **(B)** All eukaryotes (Binary Jaccard). **(C)** Plant taxa (Bray-Curtis). **(D)** Plant taxa (Binary Jaccard).

To evaluate the community structure of eukaryotes in the fecal samples of Sichuan takin across the four regions, we performed principal coordinate analysis (PCoA) based on Bray-Curtis and Jaccard distance metrics, and environmental fitting (envfit) was applied using altitude as a single environmental factor to assess its influence on the community structure (Figures 2A,B). The first principal coordinate (PCoA1) accounted for 22.21 and 9.02% of the variation in Bray-Curtis and Jaccard distance data, respectively. Samples from the RA group exhibited a more dispersed distribution, indicating variability within this group. Notably, samples from RA and RC groups largely overlapped with each other, as did those from RB and RD groups, suggesting similar eukaryotic community structures within these paired groups. However, the separation between the RA/RC and RB/RD groups was evident, indicating significant differences in eukaryotic community composition between these two clusters of regions. The results of the envfit analysis revealed that altitude accounted for 70% of the variation in the eukaryotic community composition (R^2^ = 0.70, *p* = 0.001), indicating that the relationship between altitude and community composition is statistically significant.

To further investigate dietary habits and gut parasite community differences across regions, we compared the community structures of plant and parasite taxa in the fecal samples. The results showed significant differences in plant community structure between the RA/RC and RB/RD groups, with high similarity within each cluster. Samples from the RB group exhibited a more concentrated distribution, suggesting a more uniform plant composition in this region (Figures 2C,D). The PCoA results for parasites showed substantial overlap among the four groups, indicating no significant differences in their communities (Supplementary Figure S3). Notably, while the altitude factor showed a significant *p*-value for plants, it was not significant for parasites. Additionally, the relatively low R^2^ values for parasites further suggest a weak environmental correlation with parasite community composition.

### 3.4 Community-composition analysis

**Figure 3:**
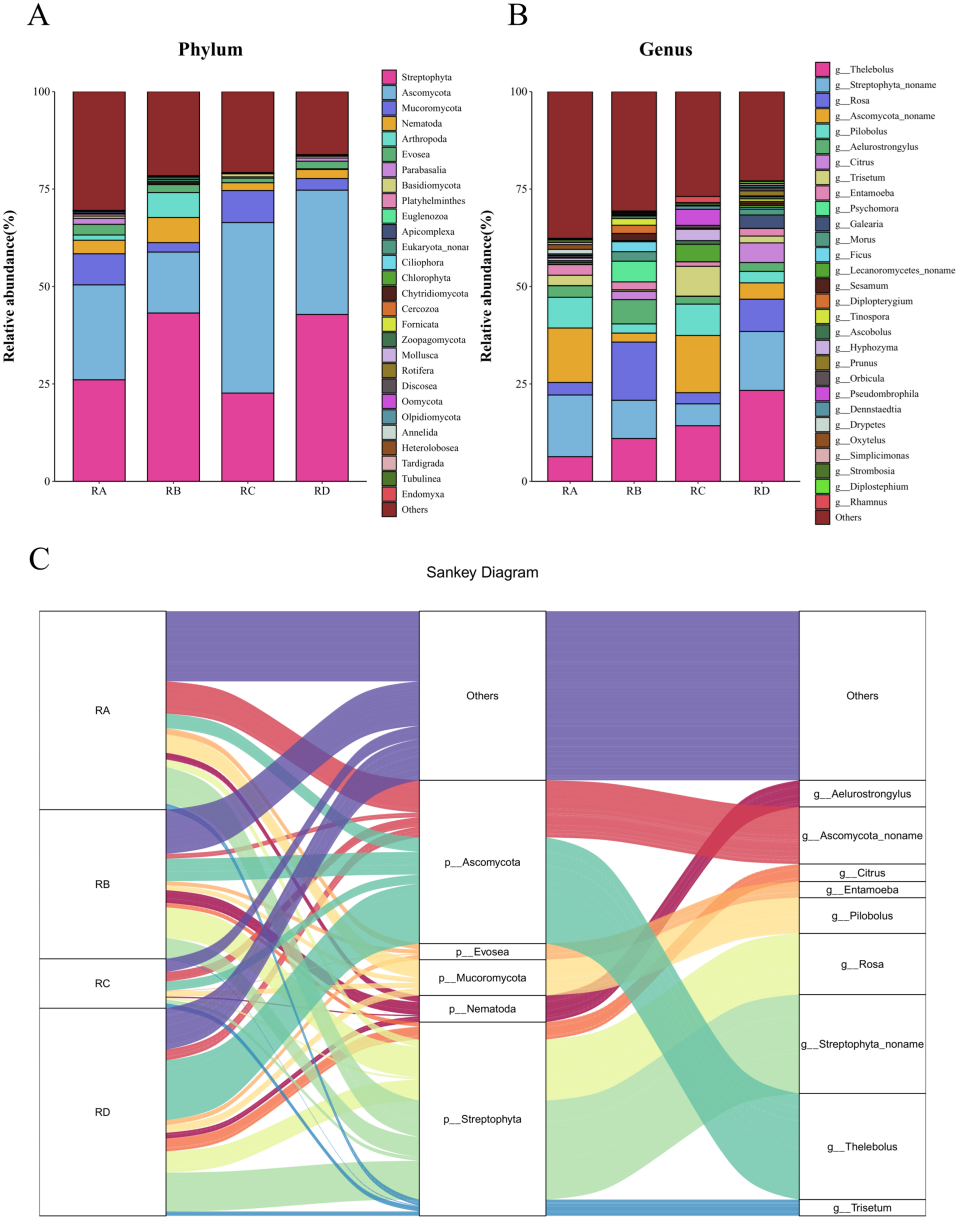
Stacked bar charts and Sankey diagram showing species abundance at different taxonomic levels. **(A)** Stacked bar chart at the phylum level. **(B)** Stacked bar chart at the genus level. **(C)** Sankey diagram illustrating taxonomic relationships.

A total of 29 phyla, 77 classes, 217 orders, 432 families, and 675 genera were identified from the fecal samples of Sichuan takin (Supplementary Tables S1, S2). At the phylum level, Streptophyta and Ascomycota dominated across all four regions, with cumulative relative abundances ranging from 50 to 80%, indicating that *Budorcas taxicolor* primarily feeds on plants from the phylum Streptophyta. Consistent with previous findings, Ascomycota was also a major fungal phylum in fecal samples from the Tangjiahe region, comprising up to 82.19% of the fungal community (10). Notably, the phylum Nematoda was also detected at relatively high abundances in all regions, with its abundance peaking at 6.44% in the RB group (Figure 3A).

At the genus level, *Thelebolus* (13.74%) and *Streptophyta_noname* (11.59%) were the most dominant genera, maintaining high abundances across all groups. Genera such as *Rosa*, *Ascomycota_noname*, and *Pilobolus* were also abundant but showed marked regional variations. For instance, the abundance of *Rosa* was significantly lower in RA (3.21%) and RC (2.86%) compared to RB (14.94%) and RD (8.28%), whereas *Ascomycota_noname* exhibited an inverse trend (Figure 3B). Among helminths, *Aelurostrongylus* was the most abundant genus (3.35%), while *Entamoeba* dominated the protozoan community; together, they accounted for over 50% of the total parasite taxa. *Aelurostrongylus* was particularly enriched in RB samples (6.22%), while *Entamoeba* was most abundant in RD samples (Supplementary Figure S4).

A Sankey diagram was constructed to illustrate the distribution and diversity of taxa from phylum to genus level across the four regions (Figure 3C). The results highlighted that Streptophyta and Ascomycota were the dominant phyla, with their abundances primarily contributed by *Streptophyta_noname*, *Rosa*, *Thelebolus*, and *Ascomycota_noname*. Within the phylum Nematoda, the high abundance was predominantly driven by *Aelurostrongylus*, a nematode that primarily infects felids and is not pathogenic to bovids such as *B. taxicolor*. This suggests that the presence of *Aelurostrongylus* in the fecal samples may result from environmental contamination, potentially through the ingestion of snails or eggs of *Aelurostrongylus* via water or plant material. For the phylum Evosea, *Entamoeba* was the primary contributing genus, accounting for most of its abundance.

### 3.5 LEfSe analysis

**Figure 4:**
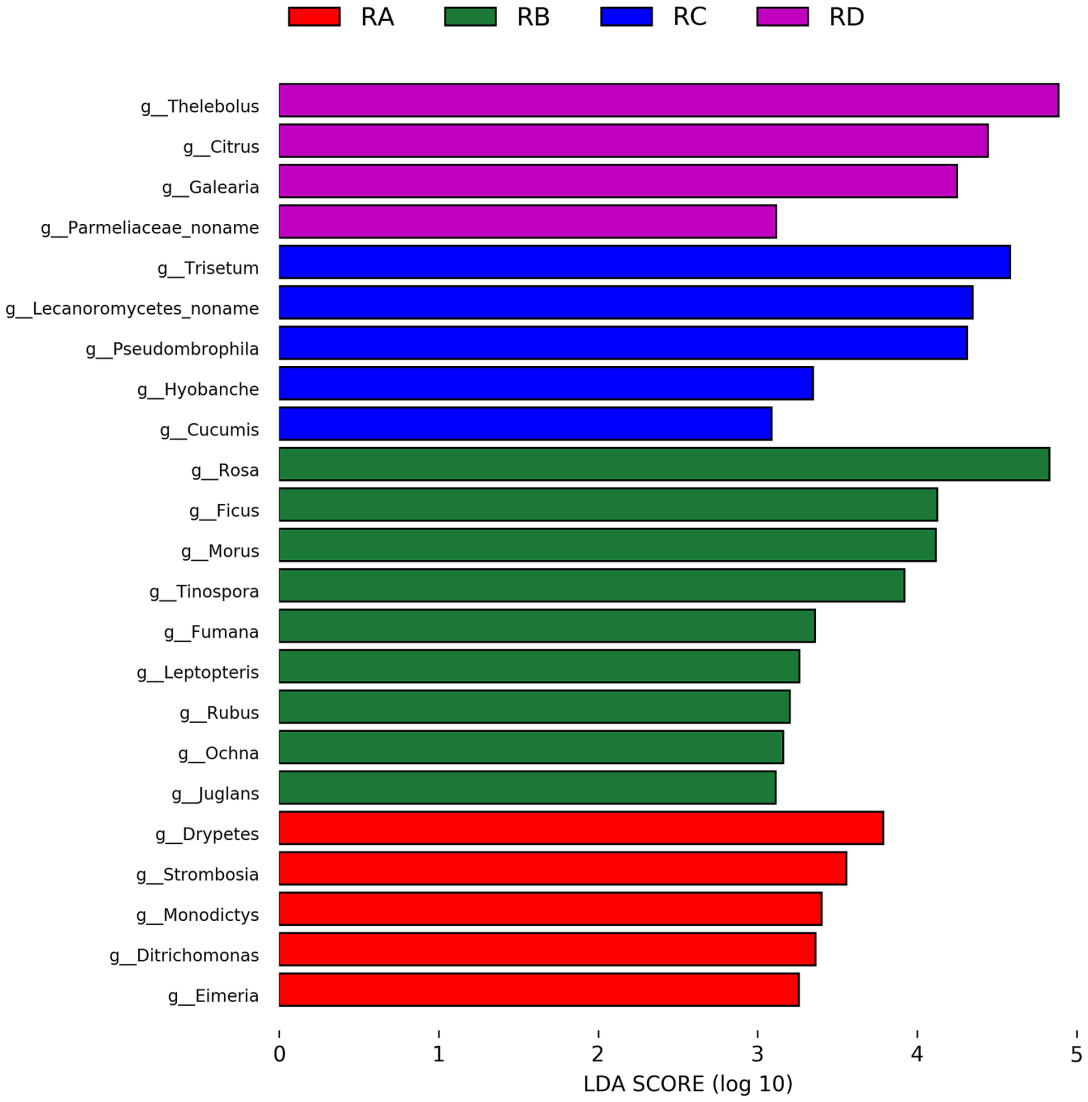
Bar chart of taxa with significant differences identified by LEfSe analysis (LDA > 3, *p* < 0.05).

We employed LEfSe analysis to identify significantly different eukaryotic taxa in the fecal samples of Sichuan takin across the four regions, selecting genera with LDA scores ≥3 for further investigation (Figure 4). The results revealed that the representative eukaryotic taxa in RB, RC, and RD groups were predominantly plants, reflecting differences in the dietary preferences of Sichuan takin and the plant diversity of their respective habitats.

Notably, in addition to plants, the RA group was characterized by the presence of *Ditrichomonas* and *Eimeria*. The pathogenicity of *Ditrichomonas* remains unclear, as no studies have directly addressed its role in disease. However, its close relative, *Tritrichomonas*, is known to cause trichomoniasis in reproductive tracts, suggesting a potential risk. *Eimeria*, on the other hand, is a well-documented pathogenic parasite, posing a significant health threat to Sichuan takin in the RA region.

### 3.6 Analysis of potentially pathogenic parasite

**Figure 5:**
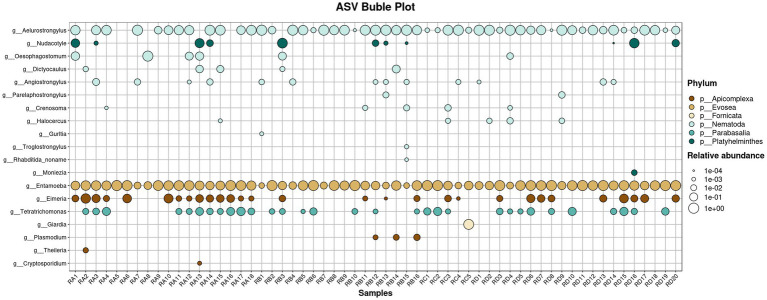
Bubble chart showing the relative abundance of potentially pathogenic parasites across 59 samples.

We screened takin fecal samples for pathogenic and potentially pathogenic helminths and protozoa, comparing their relative abundances across groups to assess potential parasitic infections (Figure 5; Supplementary Figure S5). A total of 10 potentially pathogenic nematode genera were identified: *Aelurostrongylus*, *Angiostrongylus*, *Crenosoma*, *Dictyocaulus*, *Gurltia*, *Halocercus*, *Oesophagostomum*, *Parelaphostrongylus*, *Rhabditida_noname*, and *Troglostrongylus*. Among these, *Dictyocaulus* and *Oesophagostomum* exhibited higher abundances, primarily detected in samples from the RA and RB groups. *Dictyocaulus* infects the respiratory systems of ruminants such as cattle, sheep, and deer, causing lungworm disease, while *Oesophagostomum* infects ruminants, pigs, and primates (including humans), leading to oesophagostomiasis (15, 16). Their elevated abundances suggest a potential prevalence of infections among takin populations in these regions. *Rhabditida_noname* was detected at low abundance in a single sample, possibly resulting from incidental ingestion during grazing. Genera such as *Aelurostrongylus*, *Gurltia*, and *Troglostrongylus*, which primarily infect felids, were also detected. These nematodes typically do not infect bovids. Notably, *Aelurostrongylus* was found in nearly all samples from the four regions with relatively high abundance, indicating a widespread environmental presence in Tangjiahe. This could pose significant risks to the health of felids in the reserve. *Angiostrongylus* was detected in higher abundance across multiple samples. The larval migration and persistence of this parasite can lead to tissue damage and inflammatory responses (17). Other nematodes, including *Crenosoma*, *Halocercus*, and *Parelaphostrongylus*, were found in low abundances and are not typical bovid parasites, suggesting they are unlikely to cause infections in takins. Additionally, two platyhelminth genera were identified with low abundances. *Moniezia* is a common cestode parasite in bovids, while *Nudacotyle* has not been reported to infect bovids (18–20).

The 18S rDNA sequencing data also revealed the presence of several potential pathogenic protozoa. Among them, *Entamoeba* was widely detected across the samples. This genus includes numerous parasitic and commensal species, some of which are known to cause diseases in animals. Similarly, *Eimeria*, the primary causative agent of coccidiosis, was identified with relatively high abundance across all four sample groups, indicating widespread *Eimeria* infection within the Sichuan takin population in Tangjiahe. Other protozoa, such as *Cryptosporidium* and *Theileria*, were detected at lower abundances and primarily in the RA group. Notably, *Giardia* was identified in one RC group sample with a relatively high abundance, suggesting potential freshwater contamination. Additionally, *Plasmodium* was found in several RB group samples, while *Tritrichomonas* are generally regarded as having low pathogenicity.

### 3.7 Plant-parasite correlations in the feces of *Budorcas taxicolor*

**Figure 6:**
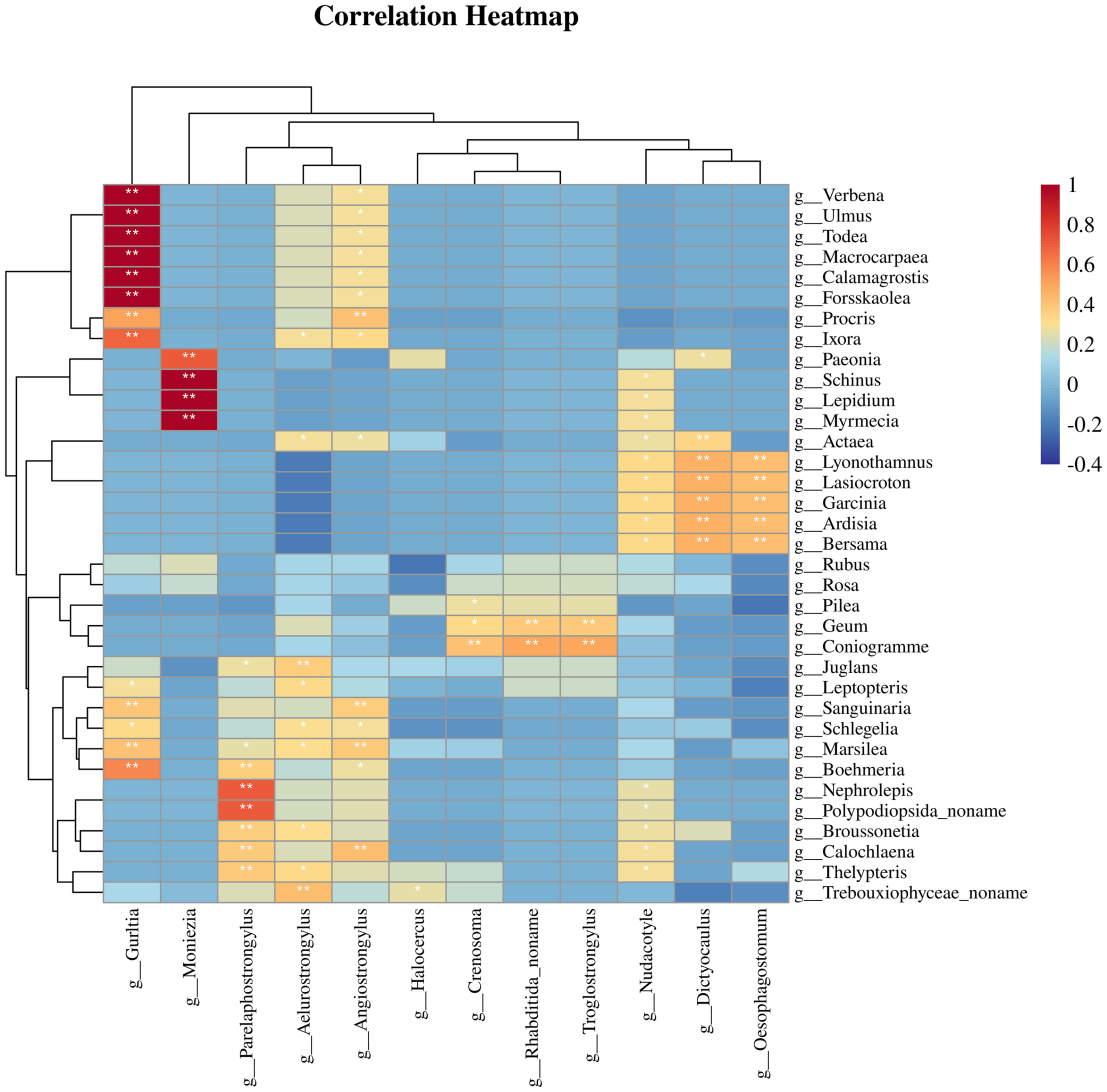
Heatmap showing the correlations between plant taxa and potentially pathogenic parasitic helminths.

To evaluate whether parasite infections in Sichuan takin were associated with their plant intake, we calculated the Spearman correlation coefficients between the abundances of potentially pathogenic parasites and plant taxa (21). The results revealed significant positive and negative correlations between specific plant and parasite genera (Figure 6). For instance, the pathogenic nematodes *Oesophagostomum* and *Dictyocaulus*, commonly found in bovids, were significantly positively correlated with certain plants, including *Ardisia*, *Garcinia*, and *Lasiocroton* (*p* < 0.01). Similarly, the tapeworm *Moniezia* showed significant positive correlations with *Lepidium* and *Myrmecia* (p < 0.01). These findings suggest that consumption of these plants may increase susceptibility to corresponding parasites. In contrast, plants such as *Rubus* and *Rosa* exhibited negative correlations with some parasites, indicating lower parasite occurrence or absence in samples with higher abundances of these plants. Overall, our results suggest a distinct correlation pattern between the distributions of plant and parasite genera.

## 4 Discussion

The Sichuan takin is a large, social mountain forest ungulate that feeds on a diverse range of approximately 160 plant species, including mosses, ferns, herbaceous plants, shrubs, and trees (6). In the Tangjiahe region, the takins are primarily distributed across altitudes ranging from 1,250 to 3,000 meters, exhibiting seasonal migration patterns. The first migration event typically occurs in November each year, when the takins move from lowland areas such as river valleys to mid-elevation regions (1,850–2,150 m) (4, 5). For this study, sampling routes were selected based on the locations of Sichuan takin signs (such as feeding marks, resting spots, and fecal matter) identified over the past five years. A total of four ecological transects were chosen, where fresh fecal samples (0–1 day) were collected across altitudes ranging from 1,100 to 2,500 meters. The takin’s migration pattern is significantly influenced by thermal conditions and changes in vegetation phenology, and the varying altitudes of their distribution across different regions are likely related to differences in solar radiation and vegetation types (22).

Alpha diversity analysis revealed no significant differences in the overall abundance and diversity of eukaryotes among the takin fecal samples across the study regions. When analyzing the alpha diversity of plants and parasites specifically, we found no notable differences in species diversity between groups. A possible explanation is that, despite differences in vegetation types and coverage across regions, the Sichuan takin exhibits habitat preference, particularly selecting areas with higher herbaceous plant coverage during winter to ensure a sufficient food supply (4). Similar habitats may influence the diversity of plants and soil-borne parasites to some extent. However, the plant abundance in the RB group was significantly higher than that in the RA group, while the parasite abundance was markedly lower than that in the RC group. The distribution of plants is influenced by elevation (23), and the lower elevation of the RB sampling sites may provide a more diverse and abundant supply of plants for takins. Similarly, although the RD group showed no significant differences compared to other groups, its Chao1 index values were consistently higher, as observed in the violin plots.

In PCoA analyses based on Bray-Curtis and Jaccard distances, significant differences in eukaryotic community composition were detected between the low-elevation and high-elevation groups. Comparisons of plant community structures in the fecal samples reflected short-term dietary behaviors and ecological adaptations of the takins (24). The plant communities in the RB group were relatively homogenous, as indicated by closely clustered samples, suggesting a simpler dietary composition for takins in this region. In contrast, the more dispersed samples in other groups likely reflected the environmental variability of these regions, corresponding to a broader range of food choices. Although there was a discernible trend in the community distribution of parasites among regions, the differences were not statistically significant. Similar results were observed when analyzing protozoa separately. Our findings suggest that the parasite community structure in takin fecal samples may be more influenced by host-specific factors and local transmission dynamics rather than environmental or dietary differences among sampling regions.

In this study, LEfSe analysis identified several taxa with significant intergroup differences, including *Thelebolus*, *Citrus*, and *Rosa*, which are predominantly associated with plants and environmental factors. Additionally, the RA group showed significant enrichment of the parasite-related taxon *Eimeria*, suggesting that its distribution may be influenced by environmental or host population differences. However, other notable parasites, such as *Entamoeba* and *Aelurostrongylus*, did not display significant intergroup differences. Bubble plots revealed that *Entamoeba* and *Aelurostrongylus* were abundant and widely distributed across samples, indicating a more uniform intergroup distribution that did not meet LEfSe’s threshold for significance. Nonetheless, their high abundance warrants further investigation.

*Aelurostrongylus* is a characteristic lungworm of felids, utilizing terrestrial mollusks (snails and slugs) as intermediate hosts. Rodents, birds, reptiles, and amphibians may act as paratenic hosts through the consumption of infected gastropods (25–27). The high abundance of *Aelurostrongylus* detected in fecal samples suggests a significant level of environmental exposure to this parasite within the Tangjiahe region. Previous studies indicate a diverse population of wild felids in the area, including a substantial population of leopard cats (*Prionailurus bengalensis*), predominantly distributed in mid-to-low elevation forests and shrubs below 3,500 m. Their activity range overlaps with that of takins, and parasite eggs and larvae in leopard cat feces may widely contaminate the environment, facilitating transmission. *Dictyocaulus*, a parasitic lungworm, causes bronchitis (verminous pneumonia) in bovids, characterized by coughing and severe pulmonary lesions, which can be fatal in severe cases (16, 28, 29). *Oesophagostomum*, particularly *O. bifurcum*, is a common parasite of livestock and animals such as goats, pigs, and non-human primates. Known for inducing nodular lesions in the intestines of infected hosts, it leads to oesophagostomiasis and is also occasionally reported in human infections (15). The high abundance of these parasites in the RA and RB groups indicates that takins in these regions may be at risk of lungworm and oesophagostomiasis, necessitating targeted health monitoring and conservation strategies. *Moniezia*, typically parasitizing the small intestine of wild and domestic ruminants, can cause reduced appetite, alternating diarrhea and constipation, anemia, and even mortality in severe cases (19, 30). Although detected in only one RD group sample, with no evidence of large-scale infection in takin populations, its presence warrants continued surveillance.

*Entamoeba* is the third leading cause of parasitic mortality globally. While most *Entamoeba* species are non-pathogenic, their high abundance in this study highlights potential health risks for hosts (31, 32). LEfSe analysis and bubble plots indicate that *Eimeria* is widely prevalent in the RA group, potentially posing negative health impacts on hosts. *Eimeria* is a common pathogen in various animals, characterized by high host specificity and the ability to cause clinical or subclinical coccidiosis. Infections typically occur at the population level via a fecal-oral route, with juvenile animals being particularly susceptible (33). Interestingly, not all RA group samples tested positive for *Eimeria*, which may be attributed to the delayed infectivity of oocysts, as external sporulation is required for transmission (34). The detection of *Theileria* in fecal samples is unusual, given that this protozoan primarily parasitizes blood cells (35, 36). Its presence may reflect indirect contamination, such as ruptured cells or environmental exposure via tick activity, necessitating further investigation into its origin and potential impacts on wildlife health. Similarly, the identification of *Plasmodium* in the RB group suggests that takins could be at risk of contracting mosquito-borne malaria. Malaria not only affects vertebrates but is also one of the most destructive infectious diseases in humans (37, 38), posing significant public health risks. It is therefore crucial to investigate the presence of mosquito vectors and the dynamics of malaria transmission in this region. *Cryptosporidium* is also a zoonotic pathogen that primarily inhabits the intestinal epithelial cells of vertebrates, causing diarrhea (39). The detection of *Tetratrichomonas* indicates its presence within the protozoan community, although its low or non-pathogenic nature (40) suggests minimal health impacts on takins. Nevertheless, its identification contributes to a broader understanding of protozoan diversity in this ecosystem.

The study identified several pathogenic parasites, most of which are primarily transmitted through the fecal-oral route, including *Eimeria* and *Oesophagostomum*. Additionally, the correlation between plants and parasites suggests a potential link between host feeding behavior and parasitic infections. *Oesophagostomum* and *Dictyocaulus*, which share similar transmission pathways through the ingestion of contaminated food or water containing infective larvae (15, 29), were significantly positively associated with *Ardisia*, *Garcinia*, and *Lasiocroton*. This suggests that these plants may harbor infective larvae, thereby increasing the risk of infection for Sichuan takin feeding on them. Conversely, the negative correlations observed between certain plants and parasites may indicate antiparasitic properties, hinting at possible self-medicative behavior (41) in Sichuan takin. For instance, *Rubus* and *Rosa*, belonging to the Rosaceae family, are known for their high tannin content (42). Tannins have been shown to exhibit significant anthelmintic activity, either by directly impairing parasite survival or by enhancing host resistance to infections (43–45). Supporting this, Sultana et al. demonstrated that three out of six compounds extracted from *Rubus niveus* aerial parts exhibited potent nematicidal activity *in vitro* (46). Similarly, Akkari et al. reported significant antiparasitic effects of *Rubus ulmifolius* fruit extracts (47). While these findings are suggestive, they are based solely on correlation analysis from sequencing data and do not establish a direct causal link between plant consumption and parasitic infection outcomes in Sichuan takin. Further studies are needed to determine whether these plants play a role in parasite transmission or whether Sichuan takin actively selects them for their potential therapeutic benefits.

Overall, this study highlights the potential impact of nematodes and protozoa on the health of the Sichuan takin population in Tangjiahe, while also examining the relationship between the dietary composition of takins and parasitic infections. This has important ecological and practical implications for wildlife conservation and health management.

## 5 Conclusion

This study provides the first comprehensive account of parasitic diversity in the endangered Sichuan takin within the Tangjiahe National Nature Reserve. The findings reveal significant differences in parasite communities across regions and identify several potentially pathogenic nematodes and protozoa, some of which, while harmless to takins, may pose risks to other species. The observed correlation between the abundance of parasitic nematodes and plant community composition underscores the potential role of plants in nematode transmission. These results highlight the importance of integrated wildlife and habitat management strategies to mitigate parasitic risks and ensure the health and conservation of takin populations.

## Data Availability

The data for this study have been published on NCBI (http://ncbi.nlm.nih.gov/) and can be found by serial number PRJNA1201350.

## Footnotes

^1^ https://www.ncbi.nlm.nih.gov/

## Supplementary material

SUPPLEMENTARY FIGURE S1 Venn diagram depicting the distribution of ASVs across different groups.

SUPPLEMENTARY FIGURE S2 Violin plots illustrating the distribution of Chao1 and Shannon indices across different groups. **(A)** Chao1 index. **(B)** Shannon index.

SUPPLEMENTARY FIGURE S3 PCoA based on Bray-Curtis and Binary Jaccard dissimilarities for parasite taxa in feces. **(A)** Bray-Curtis. **(B)** Binary Jaccard.

SUPPLEMENTARY FIGURE S4 Stacked bar chart showing the relative abundance of parasite taxa at the genus level.

SUPPLEMENTARY FIGURE S5 Bubble chart illustrating the relative abundance of potentially pathogenic parasites across different groups.

SUPPLEMENTARY TABLE S1 Sampling information table of different region.

SUPPLEMENTARY TABLE S2 The chao1 and shannon index.
