# Supplementary figures and images for: Analysis of parasite communities and potentially pathogenic parasites in wild takin (Budorcas taxicolor)
Source: Front Vet Sci. 2025 Mar 24;12:1555400. doi: 10.3389/fvets.2025.1555400 (PMC11973274; doi:10.3389/fvets.2025.1555400)

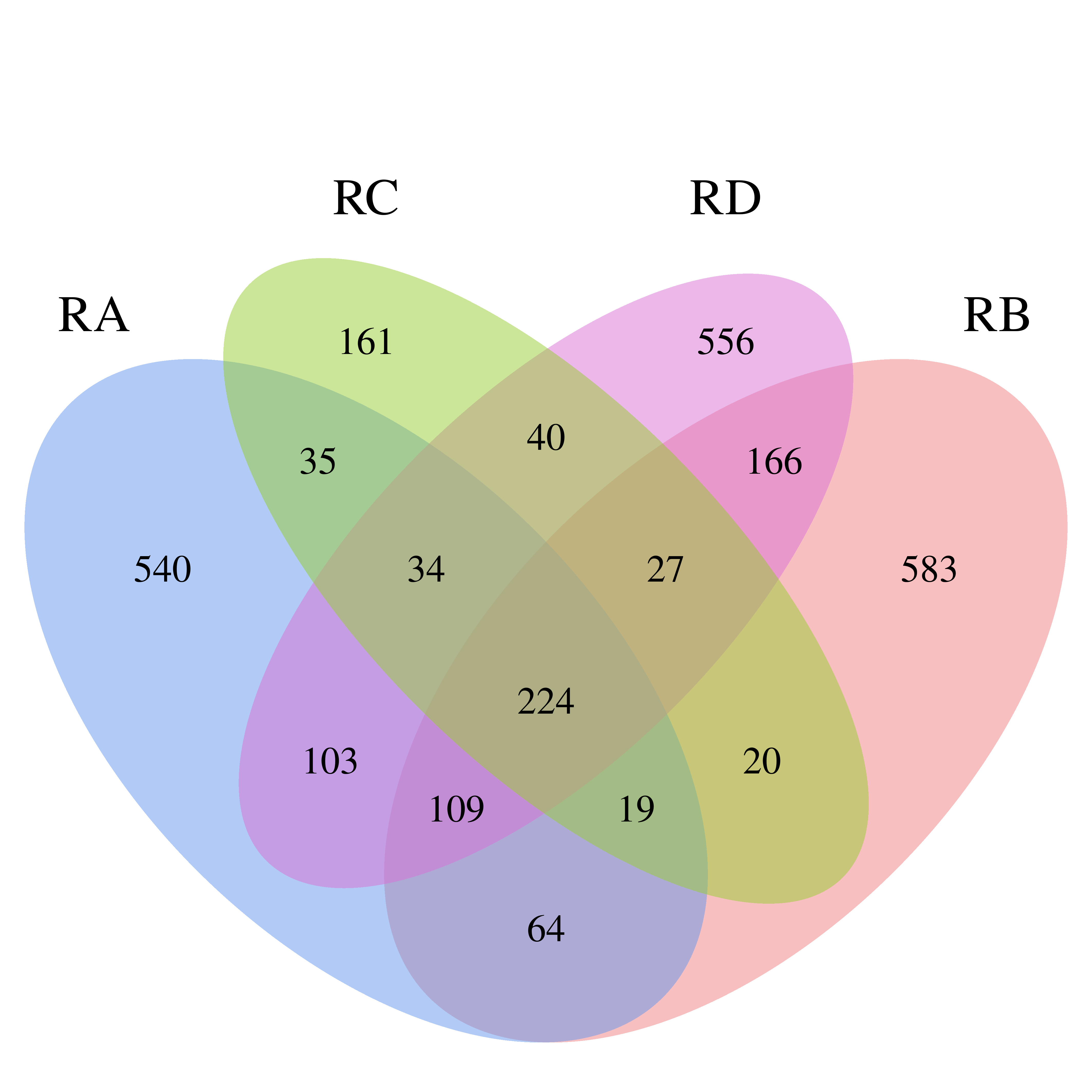

Supplement: SUPPLEMENTARY FIGURE S1 — Venn diagram depicting the distribution of ASVs across different groups. [file Image_1.JPEG]

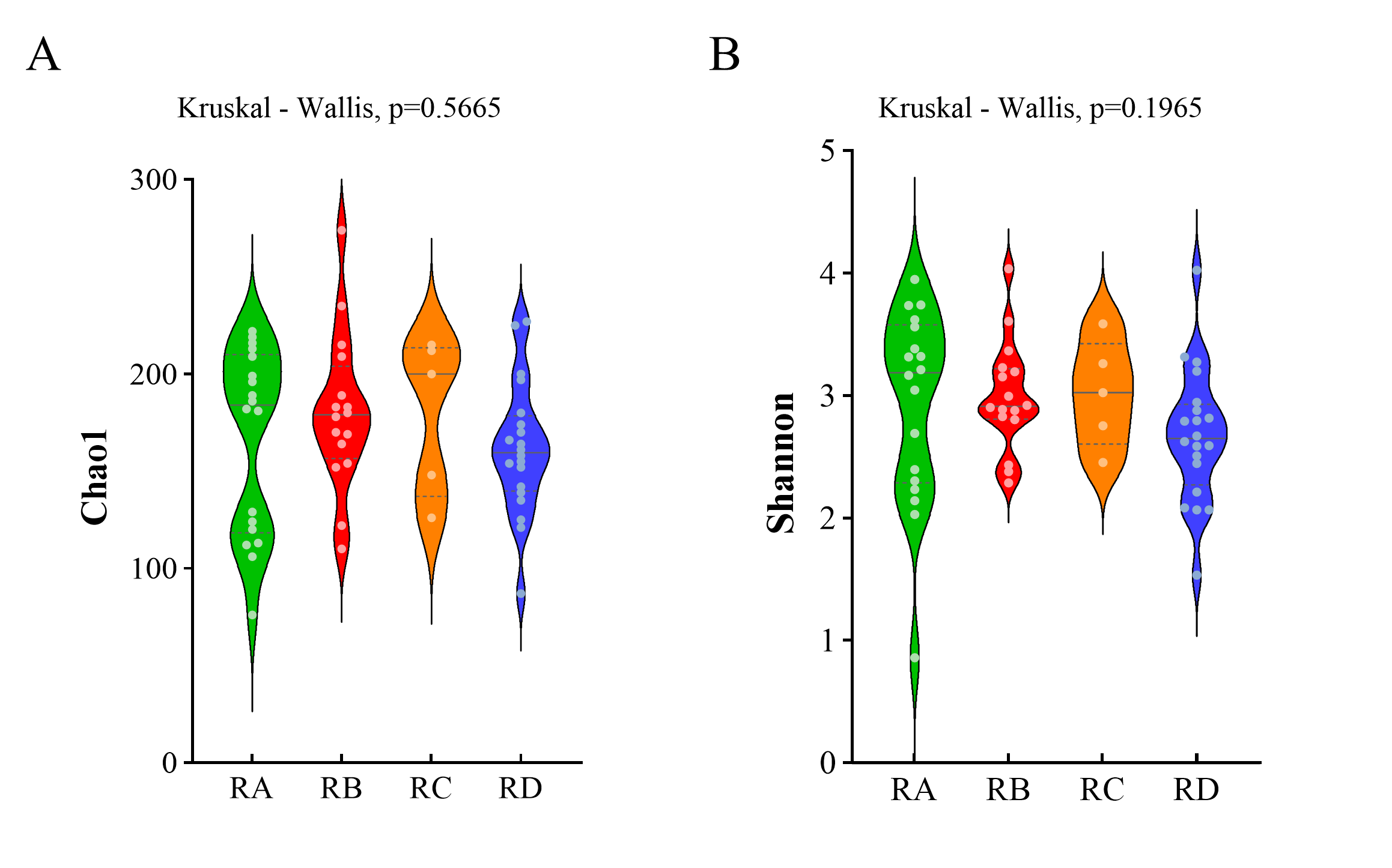

Supplement: SUPPLEMENTARY FIGURE S2 — Violin plots illustrating the distribution of Chao1 and Shannon indices across different groups. (A) Chao1 index. (B) Shannon index. [file Image_2.JPEG]

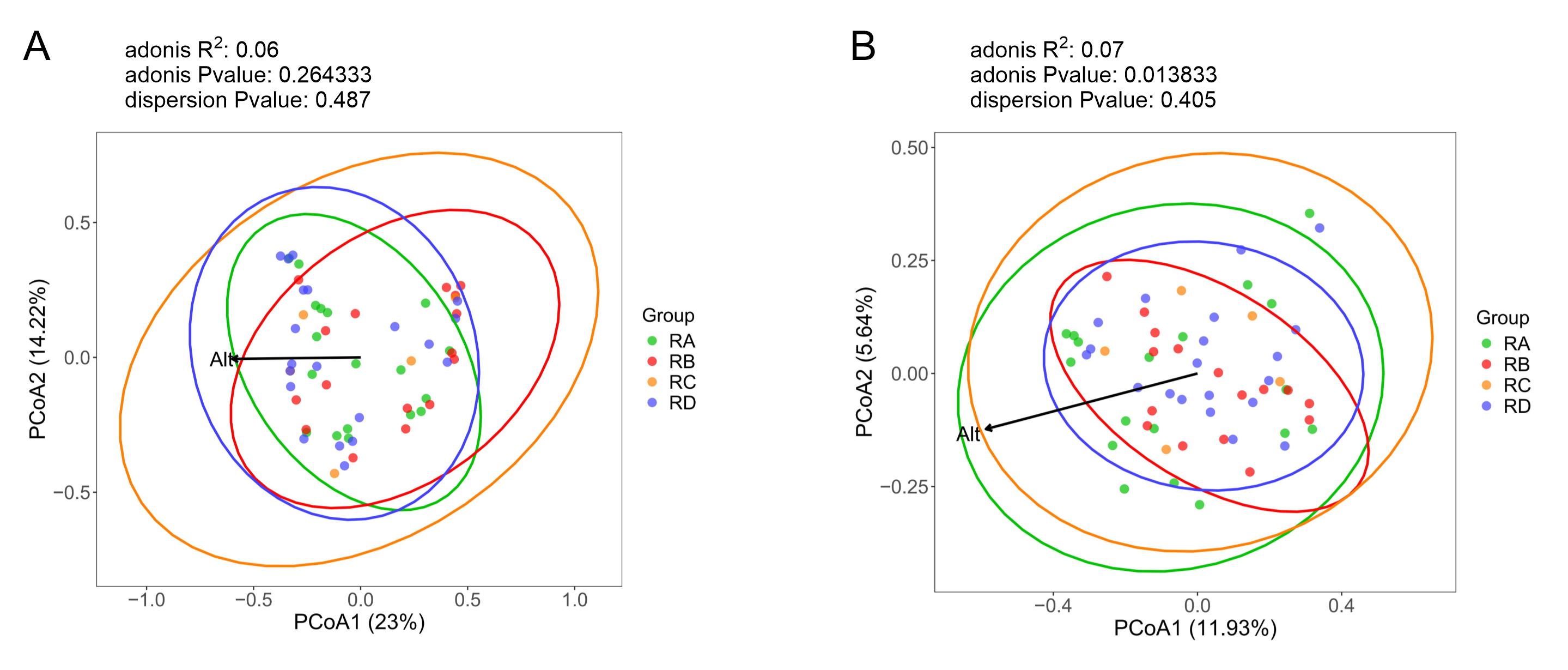

Supplement: SUPPLEMENTARY FIGURE S3 — PCoA based on Bray-Curtis and Binary Jaccard dissimilarities for parasite taxa in feces. (A) Bray-Curtis. (B) Binary Jaccard. [file Image_3.jpg]

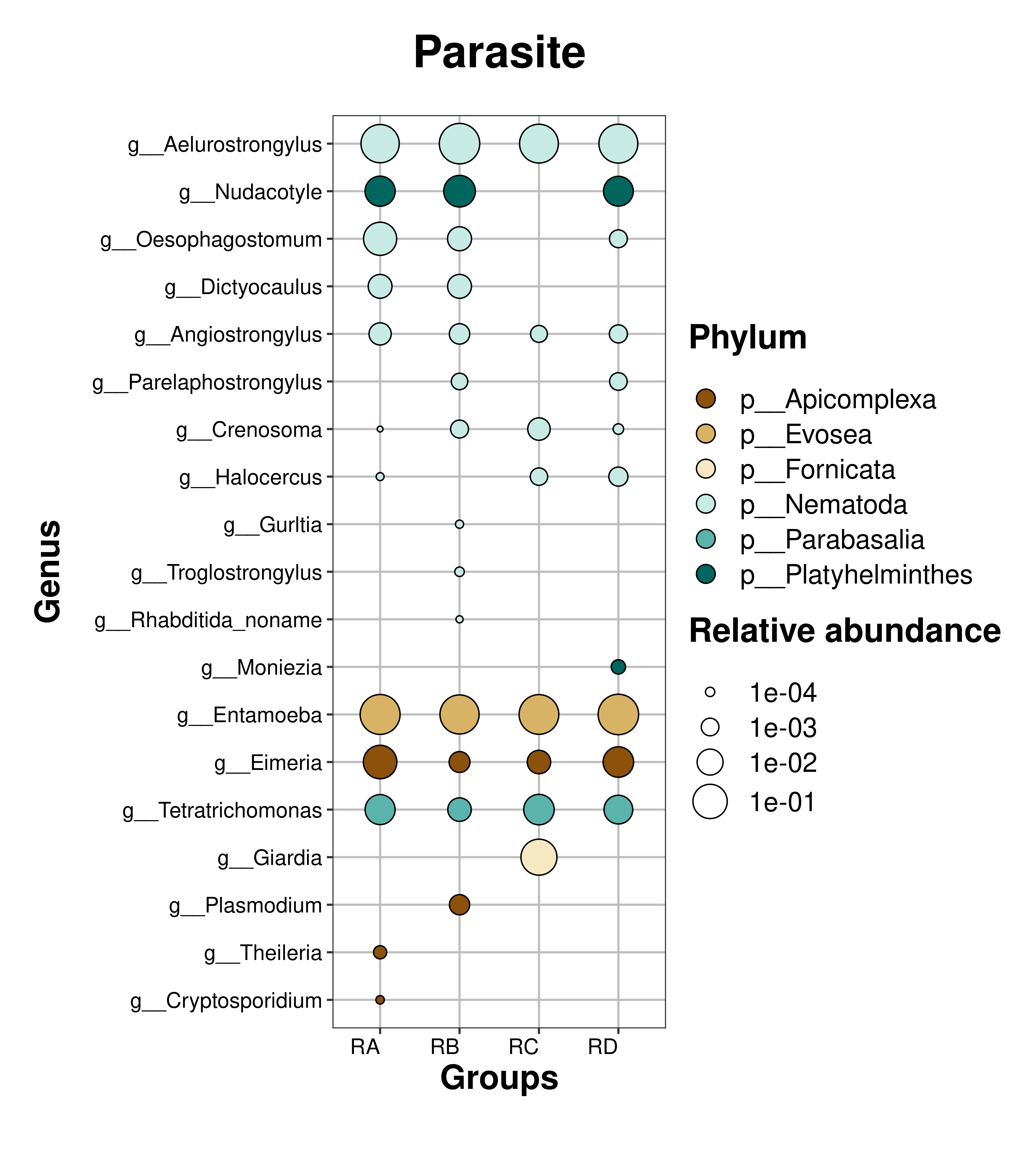

Supplement: SUPPLEMENTARY FIGURE S4 — Stacked bar chart showing the relative abundance of parasite taxa at the genus level. [file Image_4.JPEG]

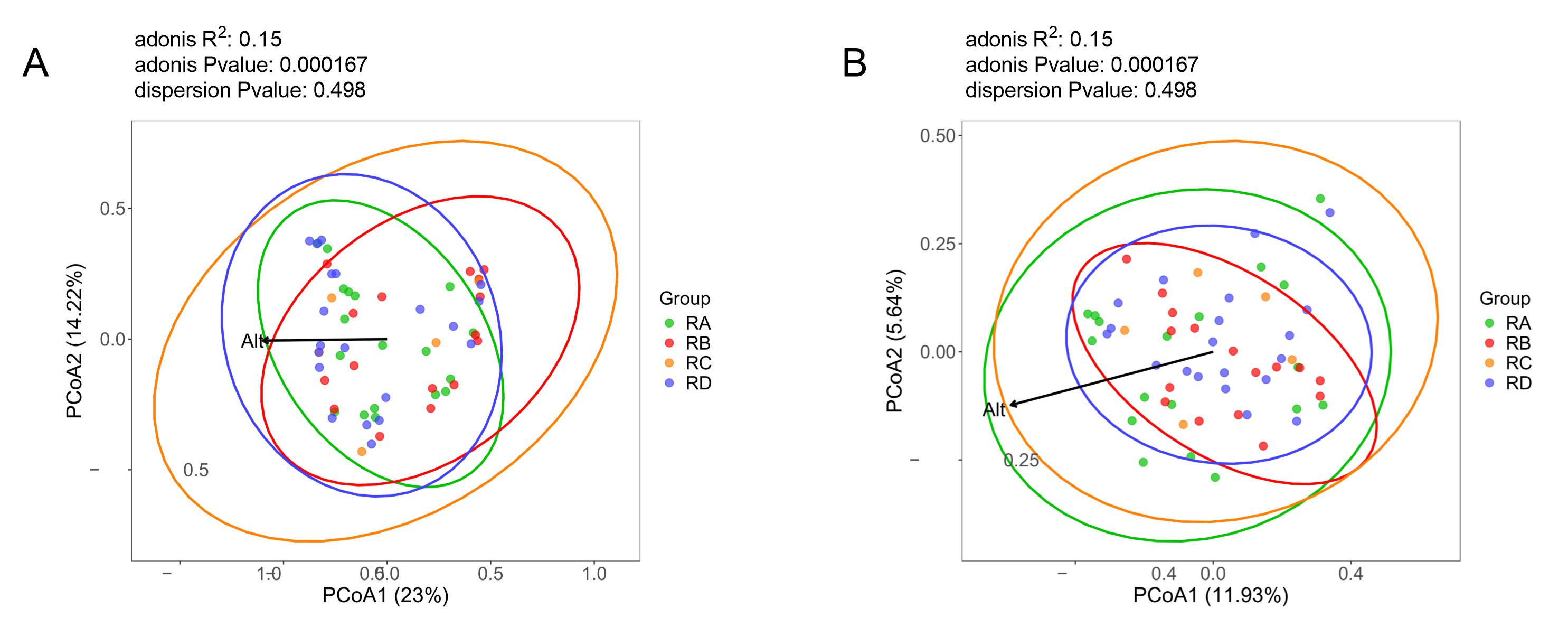

Supplement: SUPPLEMENTARY FIGURE S5 — Bubble chart illustrating the relative abundance of potentially pathogenic parasites across different groups. [file Image_5.JPEG]
